# Aquaporins and Their Regulation after Spinal Cord Injury

**DOI:** 10.3390/cells7100174

**Published:** 2018-10-18

**Authors:** Andrea M. Halsey, Alex C. Conner, Roslyn M. Bill, Ann Logan, Zubair Ahmed

**Affiliations:** 1 Neuroscience and Ophthalmology, Institute of Inflammation and Ageing, University of Birmingham, Birmingham, B15 2TT, UK; amh776@student.bham.ac.uk (A.M.H.); a.logan@bham.ac.uk (A.L.); 2 Institute of Clinical Sciences, University of Birmingham, Birmingham B15 2TT, UK; a.c.conner@bham.ac.uk; 3 School of Life and Health Sciences, Aston University, Birmingham B4 7ET, UK; r.m.bill@aston.ac.uk

**Keywords:** spinal cord injury, edema, aquaporin, water channel

## Abstract

After injury to the spinal cord, edema contributes to the underlying detrimental pathophysiological outcomes that lead to worsening of function. Several related membrane proteins called aquaporins (AQPs) regulate water movement in fluid transporting tissues including the spinal cord. Within the cord, AQP1, 4 and 9 contribute to spinal cord injury (SCI)-induced edema. AQP1, 4 and 9 are expressed in a variety of cells including astrocytes, neurons, ependymal cells, and endothelial cells. This review discusses some of the recent findings of the involvement of AQP in SCI and highlights the need for further study of these proteins to develop effective therapies to counteract the negative effects of SCI-induced edema.

## 1. Introduction–Human Aquaporins 

Water can move rapidly into and out of most cells of the human body. This passive, bidirectional transport of water may occur directly across biological membranes. However, the phosopholipid bilayer can restrict the rate of water flow depending on the properties of the cell [1]. As such, the rate of water transport can be increased through co-transport with other solutes and/or ions such as salt [2] or through water channel proteins. This type of transport is important for the maintenance of physiological homeostasis and it seems that the majority of rapid water transport occurs through aquaporins (AQPs) [3]. AQPs are a family of transmembrane (TM) channel proteins that facilitate the transcellular flow of water through biological membranes in response to changes in osmotic and/or hydrostatic pressure [4,5]. Thirteen functional human AQP isoforms have been discovered to date with ubiquitous yet differential expression across tissues of the human body [6,7,8]. 

The initial discovery that AQPs were functional water channels came from transiently transfecting AQP1 into Xenopus oocytes which, when exposed to distilled water, swelled significantly when compared with control, non-transfected oocytes. This was the result of increased AQP1 levels present within the cell membrane, which allow an influx of water to occur when the extracellular solution was hypotonic to the intracellular [9,10]. The discovery of AQPs and the development of such models has advanced our understanding of how cells and tissues regulate water flow under physiological and pathological conditions. The involvement of AQPs in many pathologies has since been increasingly brought to fruition including in cancer, epilepsy, and neurodegenerative diseases. As such, our understanding of AQPs, not only as passive facilitators of water flow but as mediators of pathological conditions, is crucial for increasing potential therapeutic targets among clinical treatments. Researchers seek to undersand more about how AQPs contribute as therapeutic targets, how they are regulated, and how we may intervene.

### 1.1. Structure of Mammalian AQPs

**Figure 1:**
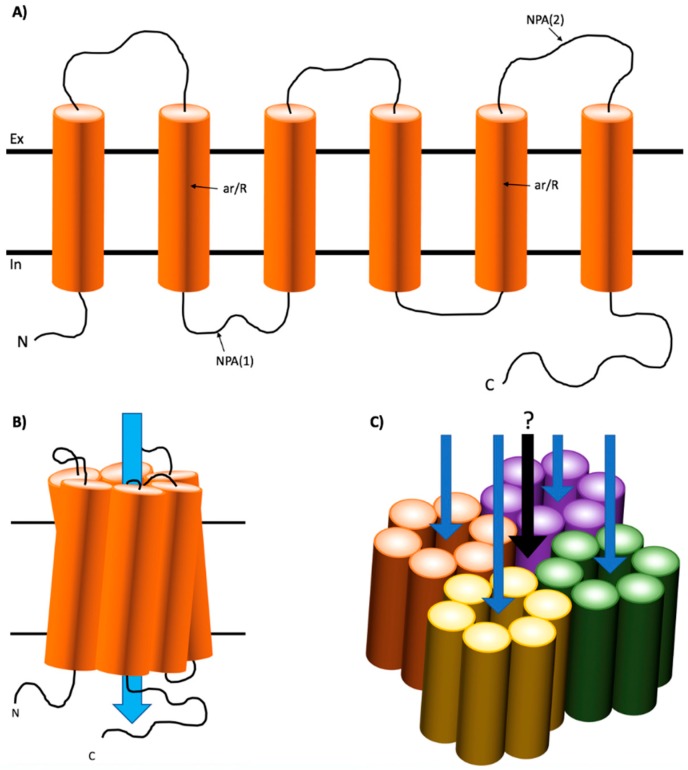
The secondary, tertiary, and quaternary structures of Aquaporin proteins. (**A**) The secondary structure of aquaporins contains six a-helices connected by three extracellular and two intracellular loops. Loops B and E contain NPA motifs and the fifth a-helices contain ar/R motifs, which is described in the text. (**B**) All six α-helices exist in a closely associated tertiary monomer structure with both ar/R motifs interacting at opposite sides of the pore and both NPA motifs interacting within the membrane. The route of water passage exists in the transmembrane pore formed through the center of the three-dimensional barrel. (**C**) AQP monomers homotetermerize and create a five-pore quaternary structure. However, the function of the central pore, formed by the space between all four monomers, remains largely unknown.

AQPs appear to exist functionally in a common homotetrameric structure [5] with recent evidence suggesting that each of the monomers behave as a functional water transport unit (Figure 1) [11]. The role of tetramerization is still incompletely defined. However, recent evidence suggests it is a requisite of the regulatory mechanism [12]. Like most TM transporters, AQPs have evolved to become highly specific for their transport molecules. AQP monomer structures exist in an “hour-glass” 3D conformation consisting of a hollow but constricted “pore” formed of six transmembrane α-helices [13,14]. The most constricted region of this pore is approximately 2.8 Å in size, which allows the continuous passage of one water molecule at a time. This is known as a single file permeation [15,16]. The electrostatic factors controlling single file permeation are conferred by the hydrophilic residues lining each pore. This includes a tightly constricted region towards the extracellular side of the pore containing an aromatic/arginine (ar/R) motif and two α-helices, which repel the passage of protons and define exquisite specificity for water molecules, and two Asn-Pro-Ala (NPA) motifs within the highly conserved pore-forming B and E loops, which interact in the center of the protein and form hydrogen bonds with water molecules to facilitate transport into the intracellular compartment of the AQP (Figure 1) [17,18,19]. These features allow AQP monomers to facilitate effective capacity and selectivity for water transport including allowing up to 3 × 10^9^ molecules of water per second across in some water-selective AQPs, e.g., AQP1, 2, 4 and 5 [4,20]. 

The tertiary structure of AQPs is considered to be of an hourglass shape with the α-helices tilting to form a barrel (Figure 1) [21]. Furthermore, subangstrom resolution-scale studies using molecular dynamics have revealed that water moves through the selectivity filter in a choreographed, pair-wise manner, which maintains electrical favorability and allows rapid transport of water through the channel [18]. Additionally, some AQPs termed ‘aquaglyceroporins’ also have selectivity for small polar molecules such as glycerol and urea [22]. These AQPs, namely AQP3, 7, 9 and 10, have an altered architecture and polarity within the constricted region that can accommodate transport of these larger molecules [22,23]. As such, the pore is slightly larger at approximately 3.8 Å and is lined with more hydrophobic residues to accommodate interactions with the additional cargo [14,24].

### 1.2. Localization and Functions of Human AQPs

There are 13 functional isoforms of human AQPs currently identified with each containing specific and localized expression patterns and functions. These isoforms have subtle differences in the primary, secondary, and tertiary structures of the proteins. The most important differences are those observed within the elements that line the pore of the channel especially the residues that offer capacitance or resistance to water and sometimes other small molecules. Since different tissues in the body require transport at different rates, the isoform localization of AQPs reflects this specificity of action. For example, different cell types in the kidney collectively express nearly all known human AQPs (AQPs 1–4 and 6–11) while the spleen only appears to express AQP9 [25]. AQPs serve different physiological functions within the body depending on their localization and isoform. Arguably the most important function of AQPs is to facilitate the general maintenance of water homeostasis within tissues [26]. This is achieved mostly though cell volume regulation (CVR) mechanisms and ensures that the osmolarity within the cell is maintained within a physiological range [27,28]. Other, more specific functions of aquaporins include, but are not limited to, water transport across kidney tubule epithelium through AQP2 [29], production of sweat and saliva through AQP 5 [30], and blood-brain/blood-spinal cord water transport through AQPs 1 and 4 [1,31]. 

### 1.3. Regulation of AQPs

Water permeation is a dynamic and fluctuating process in most tissues of the human body and, therefore, functional regulation of AQPs are critical to their activity. This is to ensure that water flow occurs at the appropriate locations and rates for each cell or tissue. Regulation of protein expression is generally the most common means of protein regulation since transcriptional rates of most genes physiologically fluctuate especially under stress [32]. This has also been demonstrated for AQPs. Activation of protein kinase C (PKC) decreases expression of AQPs 4 and 9, which can be prevented by the addition of PKC inhibitors. This suggests that their expression levels may be regulated by cellular stressors or signals that are mediated through PKC [33]. Many other studies report AQP gene expression levels being changed both via mRNA upregulation and downregulation under a wide variety of different pathophysiological conditions such as human renal and muscular diseases as well as after CNS trauma [34,35,36]. 

The most common and frequently observed method for regulating AQP activity is through conformational ‘gating’. This type of regulation was first reported in plant AQPs such as those in spinach where an intracellular loop physically barricades the intracellular pore following dephosphorylation at a local serine residue [37,38]. While this regulatory mechanism is used extensively by many biological membrane proteins, it’s unclear whether this also occurs as distinctly or as frequently in the proteins of mammalian cells. This remains a controversial topic in the field. Further to blockage gating, some studies report that AQP4 may also be gated by a physical narrowing of the channel pore that becomes too small for water molecules to pass through. Molecular dynamics studies reveal that a histidine residue in the cytoplasmic side of the pore may reorientate and interact with a nearby cysteine residue, which constricts the pore [39]. However, no biological relevance of this gating has yet been reported.

Another potential AQP regulation mechanism involves alteration of the abundance of AQPs at the cell membrane via protein trafficking. This hypothesis postulates that enhanced water flow may be the result of variable rates of translocation among AQP channels between the cell membrane and intracellular stores (short-term). Evidence for such a mechanism has been well demonstrated for AQP2 in kidney endothelium [40]. In this context, AQP2 is thought to be under the regulation of vasopressin-mediated translocation to the membrane of principal cells of the renal collecting duct, whereby vasopressin binding to its natural receptor, the vasopressin type 2 receptor (V_2_), results in a G-coupled intracellular response leading to protein kinase A (PKA) activation, AQP2 phosphorylation, and the ability to translocate to the basolateral membrane [40,41,42]. This mechanism has also been demonstrated to occur with AQP1 and AQP4. One study demonstrated through an oocyte cell-swelling assay that cyclic adenosine monophosphate (cAMP)-dependent PKA phosphorylation of AQP1 resulted in increased cell swelling due to the increased abundance of functional AQP1 at the cell surface [43]. It was suggested that this was due to direct phosphorylation of AQP1. However, the study does not indicate whether this regulates AQP function through gating or by protein trafficking. In contrast, a similar regulatory mechanism was also reported in AQP4. However, this showed decreased AQP4 permeability after PKC activation and phosphorylation of Ser^180^ [44,45].

Mechanisms for the short-term regulation of AQPs are unclear especially any differences between isoforms and tissues. Since water transport is determined passively by changes in osmolarity and tonicity, the rate of this transport seems to be mostly determined by the abundance of AQPs present at the cell membranes. Therefore, more research is needed to elucidate the exact regulatory mechanisms controlling each of the AQPs including some of which may be tissue specific as well as altered within disease or damage states. Regulation of CNS-localized AQPs will be discussed later in the context of their functions.

## 2. AQPs in the CNS–Physiological Roles

The central nervous system (CNS), which is comprised of the brain and spinal cord, is responsible for collating and processing neuronal information, interpreting its meaning, and, in some cases, coordinating an appropriate response. It is comprised of a vast number of highly specialized cells, which can be broadly categorized into neuronal cells and glial/supporting cells. These all work in tight coordination to ensure that its functional responsibilities are efficiently fulfilled [46,47]. The functionality of the CNS is very tightly coupled to the ionic and osmotic environments within the tissue and surrounding the tissue. The basic principles of neuronal conductivity rely on a balance of intra-cellular and extra-cellular concentrations of Na^+^, K^+^, and Ca^2+^ ions, which affect intra-cellular and extra-cellular osmolarity, and vice versa. Changes in osmolarity determine the flow of water into both cells and the extracellular space from the blood. Furthermore, the brain exists within a finite space, which is determined by the size of the skull. Water content within the brain directly affects intracranial pressure, which is asserted on the tissue [48]. Water levels must be effectively regulated to ensure that the brain volume does not become too high, which results in increased intracranial pressure (ICP) and leads to conditions such as intracranial hypertension (IH) [49]. 

The potential fluid-tissue interfaces that exist for exchange in the CNS are extremely restricted including those for water molecules. As such, to maintain the optimal osmotic environments, the CNS must be specialized to ensure that water homeostasis is regulated efficiently to maintain optimal functionality. One of the key specializations is the presence of specific AQPs. Transcriptional profiling of rodent genes indicates that in the healthy, uninjured mammalian spinal cord, there is an expression of AQPs 1, 4, 5, 8, and 9 across different cell types [50]. Some CNS AQPs are expressed most predominantly in distinct cell types. For example, AQP4 is expressed in astrocytes or AQP5, 8 and 9 are expressed in neurons [50,51,52]. These AQPs regulate water flow between intracellular and extracellular compartments to regulate cell volume, osmolality, and ion concentration. However, their expression levels and functions within different regions of the CNS vary and, since the blood-CNS barriers are so tightly restricted, AQPs represent the primary mechanism of mass water transport into and out of the CNS. Currently, only AQPs 1, 4, and 9 have been functionally implicated in the spinal cord and, as such, our discussion will be limited to these. However, an overview of the localization and known functions among all CNS AQPs can be found in Table 1. 

### 2.1. AQP1 

AQP1 expression has been reported at locations throughout the entire CNS in multiple cell types including ependymal cells, astrocytes, and, to a lesser degree, vascular endothelial cells [52,53,54]. The most abundant expression of AQP1 is at the apical membrane of choroid plexus epithelium (CPE) ependymal cells in the brain–the tissue residing in the lateral, third, and fourth brain ventricles, which is responsible for the production of cerebrospinal fluid (CSF) [55,56,84]. It is the role of AQP1 to facilitate rapid and regulated water transport between the CPE and the ventricles in order to produce CSF. When AQP1 is knocked down in mice, there is an 80% reduction in the water permeability of the luminal CPE membrane but only a 25% reduction in CSF production [31]. This suggests two things. First, despite the passive nature of AQPs, their expression levels should not be interpreted directly as being representative of absolute water flow. This is because AQPs do not actively control water flow. This remains under the control of the local osmotic pressures they are exposed to. Second, there may be other non-AQP1-dependent mechanisms facilitating CSF production in the CFE potentially via paracellular transport. CSF release through AQP1 is thought to be very important for maintaining physiological ICP since AQP1 knockout mice exhibit 56% lower ICP than controls [31].

In addition to ependymal cells, a small population of AQP1 appears to be expressed within some neuronal and glial cells in the brain. Immunolocalization studies evaluating the expression changes of AQPs following the CNS insult have revealed that, even in sham controls, a small level of AQP1 expression may be observed within the rat parietal cortex. While the precise physiological role of AQP1 in these neurons remains unknown, evidence suggests that their upregulation in traumatic brain injury (TBI) models is involved in pain processing and, therefore, perhaps their role is more pathological [57,85]. Furthermore, while AQP1 is not detected in astrocytes in the healthy brain, it appears to be upregulated within astrocytomas in humans [58,59] and also in astrocytes following a subarachnoid haemmorage and following brain stab injury in rats [60], which is an effect that has been shown by cell culture studies to probably occur in response to hypertonicity via a hypertonicity-response element in the AQP1 gene [86,87]. 

### 2.2. AQP4 

AQP4 is expressed in high levels across the brain, spinal cord, and retina as well as in some peripheral nervous tissue with the highest expression levels detected in the cerebellum and spinal cord grey matter [50,64]. Furthermore, its expression has been localized in multiple cell types including astrocytes, endothelial cells, and some smaller populations of neurons [50,65]. Its functions are thought to include aiding astrocyte signalling, regulation of local ion homeostasis, and CVR (cell volume regulation) [88,89].

#### 2.2.1. Astrocytes 

**Figure 2:**
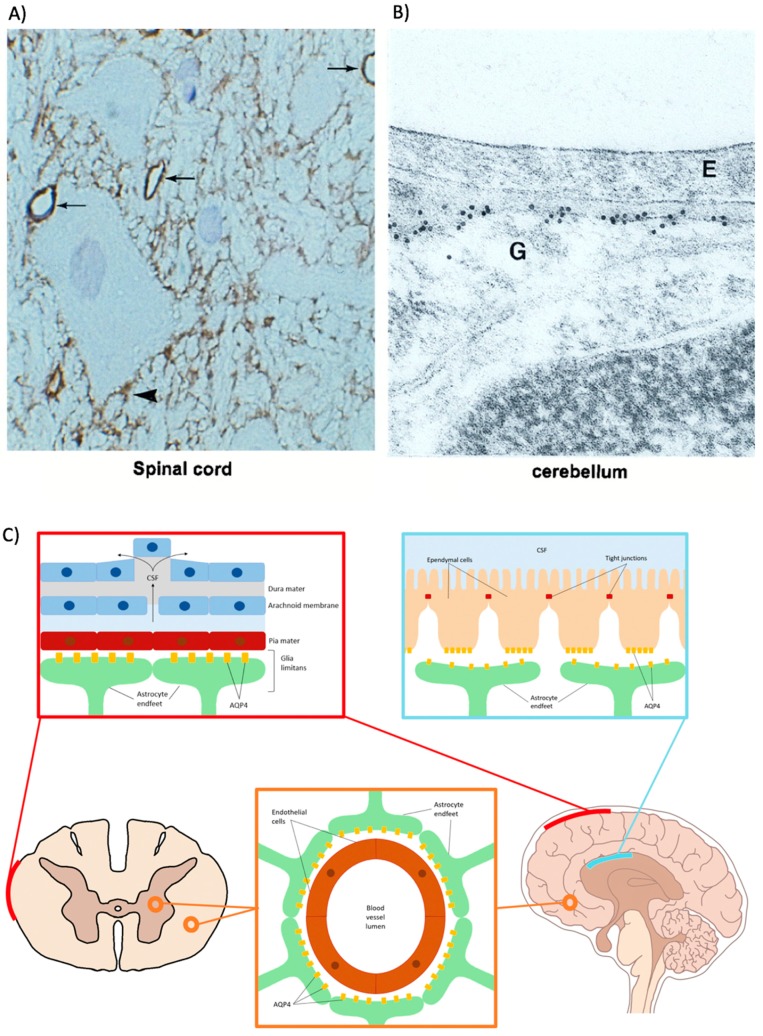
Localization of aquaporin-4 in the brain and spinal cord. (**A**) Immunogold labelling of AQP4 in thin cryosection of spinal cord, which highlights extensive staining surrounding capillaries (arrow) and neurons (arrowhead) (×670.) (**B**) Immunogold labeling of AQP4 in ultrathin sections labeled for endothelial cells (E) and glial endfeet processes (G) (×72,000.) (**C**) Schematic representation of the localization of AQP4 in CNS tissues. AQP4 is located within astrocyte endfeet processes surrounding blood vessels in both brain cortical tissue and spinal cord white and grey matter (orange panel). It is also located at the outer membrane glial surfaces of both organs (red panel). In the brain, AQP4 is also present on ependymal cells lining brain-CSF interfaces (blue panel). Figures from A and B taken from Rash et al., 1998.

The most prominent location of AQP4 in the CNS is within the membranes of polarized astrocyte perivascular end feet surrounding blood-CNS and CSF-CNS interfaces in the brain and spinal cord (Figure 2) [65,67]. Accordingly, AQP4 expression strongly co-localizes at this site with glial markers such as glial glutamate transporter-1 (GLT-1) and glial fibrillary acidic protein (GFAP) [68]. AQP4 becomes confined to endfeet membranes due to the interaction of this membrane region with other cell types. The relative level of immunoreactivity of AQP4 in the membrane compartment of astrocytes significantly increases in the presence of agrin, which is a membrane-bound proteoglycan found on neurons and endothelial cells [90,91]. Furthermore, α-syntrophin knock-out mice display significantly reduced endfeet-localized AQP4 than wildtype controls, which is supported by other studies demonstrating that astrocytes co-cultured with endothelial cells also decrease their membrane-to-cytosol AQP4 expression when α-syntrophin is knocked down [92,93]. AQP4-expressing astrocyte endfeet can be found throughout many locations of the CNS with the highest abundance surrounding the blood vessels within the superficial laminae of grey matter in the spinal cord and at the pial membranes at the surface of the brain. AQP4 is also found to a lesser degree in astrocyte end feet surrounding the glial-limiting membrane, myelinated neuronal fibers, and the central canal of the spinal cord [50,61].

The identification of AQP4 at these locations probably relates to the identification of crystal-like intramembranous protein ‘assemblies’ that were previously observed in the brain and termed orthogonal arrays of particles (OAPs) [66,69]. AQP4 was subsequently confirmed as the OAP protein constituent due to their disappearance in *Aqp4^−/−^* knockout mice [70]. These AQP4 assemblies are built from M1 and M23 isoforms of the protein monomer and the relative abundance of these isoforms directly affects the polarization of AQPs to astrocytic endfeet [71]. Higher M23:M1 ratios favor larger OAP formations, which increases the water permeability of the endfeet [94]. Collectively, the tissue and subcellular localization of AQP4 in the CNS alludes to the primary functions of AQPs in the CNS. For example, the strong expression of AQP4 around the brain and the spinal cord astrocyte-fluid interfaces strongly suggests that AQP4 has an important role in regulating water flow into, and out of, the CNS. 

Many of the physiological functions of AQP4 have been revealed by using genetically modified mice models exposed to varied pathological conditions [72]. One of the earliest examples of this was in *Aqp4^−/−^* mice models, which exhibited vastly reduced levels of post-ischemic edema following an induced ischemic stroke [73]. Furthermore, AQP4 deletion within glial cells in mice resulted in a 31% decreased uptake of water into the brain following hypo-osmotic stress. However, the overall brain dry mass did not change, which suggests that the role of AQP4 at the blood-brain (and potentially blood-spinal cord) barriers exists pathophysiologically and mediates water absorption in hypo-osmotic conditions [74]. More specifically, AQP4 has been demonstrated to regulate cell volume homeostasis by maintaining extracellular solute clearance. For example, primary astrocytes demonstrate a response to hypo-osmotic stress by rapidly increasing cell volume, which is a response that was absent from identical mouse astrocytes in which AQP4 is knocked down (*Aqp4^−/−^*) [75]. Additionally, AQP4 is also expressed at other astrocytic locations including perisynaptically but to a significantly lower density [65]. It is documented that high-frequency activation of excitatory synapses results in a reduction in the extracellular space surrounding the synapse [76]. However, in *Aqp4^−/−^* mice, this volume reduction is enhanced [74]. Therefore, AQP4 is thought to regulate perisynaptic volume by increasing water efflux in response to hyperosmolality, which occurs due to high-frequency activity and the resulting water absorption through the Na^+^/K^+^/2Cl^−^ cotransporter 1 (NKCC1) into astrocytes. 

AQP4 is also present with a non-polarized distribution within fibrous astrocytes of the spinal cord white matter and optic nerve and also within Muller cells within the retina [61,77,95]. In the spinal cord, white matter astrocytes expressing AQP4 are present throughout the whole of the membrane including along processes and at the cell body. These processes extend radially across the white matter regions—from borders of grey matter to the outer glial limitans [78]. More specifically, these processes have been found to envelope myelinated neuronal fibers [61]. However, the physiological and pathophysiological relevance of AQP at this localization remains currently unknown.

Most of the studies investigating roles of astrocytic AQP4 tends to focus on either the brain or the spinal cord. At present, the translation of knowledge of the AQP4 location to physiological function is not conclusive. However, due to the highly similar cellular and sub-cellular distribution seen at different CNS locations, it is expected that the same or similar functional mechanisms of AQP4 occur throughout the whole CNS. 

#### 2.2.2. Ependymal Cells

AQP4 is also observed within the basolateral membrane of the ependymal cells surrounding brain-CSF barriers [66]. This suggests that AQP4 has a physiological function in maintaining water transport between CSF and CNS parenchyma. Some studies suggest that ependymal AQP4 is involved in transependymal CSF flow and/or reabsorption, which, when disrupted as in *Aqp4^−/−^* mice, results in sporadic hydrocephalus [79]. However, more research is required to clarify the role. Ependymal AQP4 expression has been notably observed in the medial habenula (HB) of the brain, which is a region involved in many cognitive processes such as mood and anxiety regulation [64,96].

### 2.3. AQP9

In the normal spinal cord, AQP9 is expressed in astrocytes, catecholaminergic neurons, and endothelial cells of sub-pial blood vessels [50,62,82,83]. AQP9 is localized to the plasma membrane and facilitates the flow of water, glycerol, monocarboxylates, and urea.

## 3. Spinal Cord Injury and Oedema

A spinal cord injury (SCI) can be broadly categorized into being of traumatic or non-traumatic origin. The former is the result of external physical insult (for example, a car accident) and the latter is the result of a much more diverse range of internal insults (for example, a tumor) [97]. The incidence of SCI is not easily defined probably due to the highly varied nature of the condition and the complications with a guaranteed diagnosis. A recent world-wide review of the literature reporting SCI incidence rates suggests that, in developed countries, the rates range between 13.1 to 163.4 per million incidents of SCI and, in non-developed countries, the rates range between 13.0 to 220.0 per million [98]. According to the World Health Organization (WHO), patients suffering from SCI have a much higher incidence of depression compared to the overall population estimates and are up to five times more likely to die prematurely [99,100]. Furthermore, SCI has a significant economic impact both on the individual and on the society with economic consequences on top of the costs of personal care [99,101,102]. As such, research is absolutely vital to investigate how spinal cord injuries manifest with such devastating functional consequences and vital for creating interventions that can minimize damage and subsequently ease the socioeconomic burden of SCI. 

Edema is a clinically important feature in the progression of SCI that causes secondary damage through physical contortion and/or obstruction of nerve fibers and vasculature that were, otherwise, uninjured by the primary damage [103]. Shortly following injury to the human spinal cord, there is a significant increase in the amount of water accumulating in the parenchyma of the damaged region [104]. This water, termed ‘edema’ forms within the acute phase of secondary injury pathogenesis and may persist for weeks. It is a vital element of SCI pathology since some studies report that the severity of the edema (generally the size and location of the swelling) is strongly correlated to the clinical outcome especially in terms of motor recovery [104,105]. Evidence shows that the edema formation begins within minutes from the epicenter of the primary injury and develops outward into a larger fluid-filled cavity within 48 hours and may persist for up to 14 days post-injury [106]. While the exact molecular mechanisms facilitating edema formation are not clear, it is thought to be a consequence of both the swelling of neurons and astrocyte end-feet (cytotoxic edema) coupled with the disruption and leakage of the blood-spinal cord barrier (BSCB) (vasogenic edema). 

Cytotoxic edema is more prominent in astrocytes than in neurons due to their sensitivity to K^+^ levels for clearance [107]. It happens as a response to a combination of local inflammatory mediators, ATP deprivation, and the local release of arachidonic acid produced via membrane lipid metabolism [108,109]. Collectively, these factors influence dysfunction of ATP-dependent Na^+^ K^+^-ATPase pumps, which drive excess Na^+^ down its electrochemical gradient and cause ionic imbalance [108,110]. To counter this, an excess of Cl^−^ ions is then driven into the cell to electrically neutralize it, which is followed by the influx of water into astrocyte endfeet. This results in cell swelling [111]. AQP4 is considered to be the mediator of this pathogenic water influx (which will be discussed later). 

Cytotoxic edema creates a premorbid driving force leading to ionic oedema, which is a process whereby highly concentrated extracellular ions (e.g., Na^+^) are altered in their normal concentration gradients. This secondarily affects the driving force for secondary molecules (e.g., Cl^−^). This dysregulation then modulates the transendothelial concentration of Na+ where the blood concentration of Na^+^ is significantly higher than that of the parenchymal concentration, which creates a new Na^+^ gradient and causes a water flux into the parenchyma across the intact BBB. This is the first stage of CNS tissue swelling [112]. The relative osmotic imbalance across the blood-CNS barrier then creates a gradient whereby water flux favoring the parenchyma occurs and the capillaries (and potentially larger vessels) now behave as though fenestrated, aiding the formation of permeability pores and resulting in vasogenic edema. 

Vasogenic edema describes the disruption of BSCB integrity where molecules that are normally highly restricted in their passage across this barrier can pass more freely such as water, glucose, and some plasma proteins. It may be observed as quickly as 90 s following the primary injury and can persist as late as two weeks in rat models [113,114]. Collectively, cytotoxic and vasogenic oedema are both critical and associated processes. Even though cytotoxic edema alone may not necessarily produce mechanical or pressure-induced impact to the spinal cord, it may intensify the premorbid state of the tissue and favor the formation of vasogenic edema.

Edema causes exacerbation of the primary injury by raising intrathecal pressure within the spinal cord, which causes further damage by reducing blood flow, leads to hemorrhages, BSCB disruption, and, subsequently, further cell death [115]. However, since edema is often a quick-onset and long-lasting feature of SCI, it remains somewhat resistant to interventions, which makes it a great challenge to overcome or prevent clinically. As such, understanding the causes and molecular mechanisms involved in the formation of edema after acute SCI is important for developing ways of attenuating its development in order to improve or abolish the adverse clinical outcomes resulting from secondary damage. 

The mechanisms of edema formation in SCIs are associated with dysregulation of water transport, which leads to the swelling of astrocyte end feet and the formation of fluid-filled cavities. This indicates that water homeostasis and the proteins involved in water transport may contribute to the resulting pathologies and, as such, may offer themselves as therapeutic targets to prevent the secondary damage elicited from edema. The exact role of AQPs in edema pathogenesis remains controversial due to published studies reporting conflicting results about the role of AQPs in causing and resolving different types of edema [82,116,117,118,119,120,121]. It is clear that AQPs play important roles in both phases of edema in the spinal cord and further studies are required to investigate the exact mechanisms associated with AQPs and their contribution to both of these processes. Herein, we will discuss the available information on AQP and their role in water regulation after SCI. 

### 3.1. AQP1 in SCI 

AQP1 levels have been shown to significantly increase under a broad range of neuropathological conditions such as Alzheimer’s, Creutzfeldt-Jakob disease, traumatic brain injury (TBI), and SCI [63,122,123,124]. In uninjured spinal cords, AQP1 expression is predominantly localized to neuronal fibers and ependymal cells. Following SCI, not only did expression increase in these cells, but also in astrocytic cell bodies and with elevated levels persisting for up to 11 months post-injury [124]. This persistent and multicellular localization suggests the possibility that AQP1 contributes to different pathological processes after SCI. Extracellular hypertonicity did not affect AQP1 unlike AQP4 that increases robustly as a result of hypertonicity. However, oxidative stress significantly increased AQP1 protein expression while the antioxidant melatonin suppressed this AQP1 upregulation [124]. Since AQP1 is expressed after hypoxia in endothelial cells play a role in angiogenesis and cell migration [116,125], AQP1 was postulated to have a similar function in astrocytes of the spinal cord or in axon elongation via water uptake [124]. In support of this assertion, AQP1 was found colocalized with the axonal growth marker GAP43. However, such sustained and persistent upregulation of AQP1 may enhance neuronal/axonal swelling [117] and may result in overproduction of CSF by ependymal cells or fluid-filled cysts after SCI [36,124]. Upregulation of AQP1 after SCI was found in reactive astrocytes surrounding the lesion site and, although the role of this response is not clear, it suggests that AQP1 may facilitate astrocyte migration to the lesion site.

### 3.2. AQP4 in SCI

AQP4 is the most well-characterized AQP after SCI. AQP4 protein levels appear to change biphasically after SCI, starting with an immediate decrease between onset and three days post-injury, which is followed by a significant increase seen as late as nine months post-injury [36]. In ischemic stroke models, *Aqp4^−/−^* mice exhibited 35% less cerebral edema when compared to wild-types (WT) with significantly reduced astrocyte endfoot swelling [73]. Further studies investigating SCI have also yielded similar conclusions whereby the inflammatory mediator IL-6 induces a HMGB1-mediated signaling cascade resulting in the increased expression of AQP4 in astrocyte endfeet and a subsequent increase in cytotoxic swelling [118]. In *Aqp4^−/−^* mice, there is also reported functional and pathological improvement after compression SCI in which mice demonstrated significantly higher motor scores and decreased edema than their WT counterparts for up to 14 days post-injury [119]. 

Collectively, studies focused on the roles of AQP4 in cytotoxic edema suggest that AQP4 is a mediator in the formation of cytotoxic edema in the CNS. However, more recently, a study showed that inhibition of AQP4 by TNG-020, which is a direct AQP4 inhibitor, following SCI reduces spinal cord water content and AQP4 expression cumulatively with the dose of the inhibitor [80]. This suggests that AQP4 also has a role in the formation of water accumulation involved in vasogenic edema. However, the precise mechanistic link has not yet been identified. In contrast, other studies focused on vasogenic edema, which resulted from BBB/BSCB dysfunction, demonstrating that AQP4 is crucial for facilitating the clearance of water [81]. 

In *Aqp4^−/−^* mice, it was observed that vasogenic edema, as determined by water content, was significantly higher up to 28 days after compression SCI when compared to control levels, which was not observed in the wild-type (WT). Furthermore, these *Aqp4^−/−^* mice also appeared to have a poorer functional recovery following SCI than their WT counterparts [120]. Another study showed that when *Aqp4^−/−^* mice were subject to transectional SCI, the water content within the injured half of the spinal cord was significantly higher than the water content within the uninjured half of the spinal cord in *Aqp4^+/+^* mice three days post-surgery. This suggests that knockdown of AQP4 enhances edema even in acute stages of SCI pathogenesis [121].

It is important to consolidate and resolve the differences observed for the roles of AQP4 between cytotoxic and vasogenic edema since both of these elements have been demonstrated to occur following human traumatic SCI [113,126]. Despite the fact that cytotoxic edema itself is not thought to cause the physical contusion that results in secondary injury, it is thought to be the ionic driving force that leads to vasogenic edema and the build-up of fluid-filled cavities that cause secondary damage [127]. As such, the traditional categorization of edema into “cytotoxic” and “vasogenic” is being increasingly considered as an oversimplification of the two sides of a connected process. 

The conflicting evidence also suggests that, in addition to the role of AQP4 in different types of edema, there must also be consideration about the type of the experimental model used since different models reflect different types of human SCI including both the severity and the pathophysiological processes that occur. For example, transactional laceration SCI likely results in significantly larger amounts of vasogenic oedema due to the direct compromise of BSCB while contusion SCIs likely spare the BSCB directly, which implies that they occur through different processes [128,129]. Furthermore, there is a time element to be considered, since *Aqp4^−/−^* mice examined shortly after injury appear to have reduced edema and better behavioral functionality than those examined later. This suggests that the role of AQP4 following SCI may be biphasic, having an edema-forming role immediately following injury, followed by a resolution role in later stages. Perhaps this functionally biphasic phenomenon of AQP4 is a result of it having two different roles. The pathological role of causing edema by encouraging cytotoxic and/or vasogenic oedema is followed by the physiological role of maintaining ionic homeostasis by regulating water flow. 

**Figure 3:**
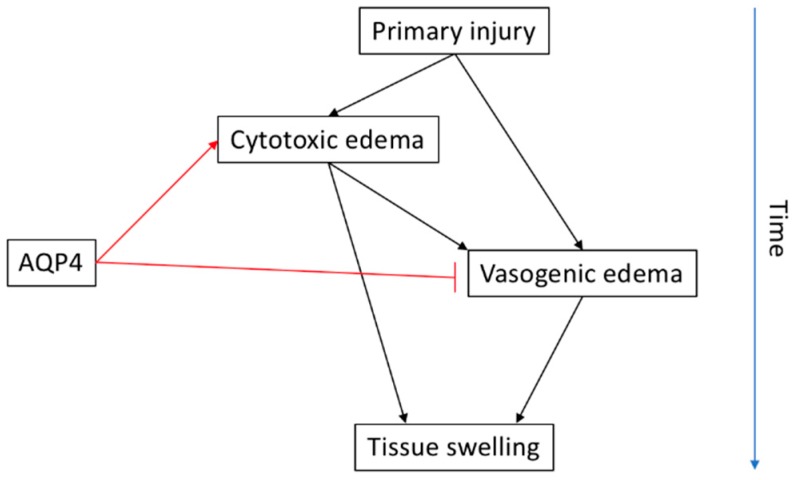
The proposed relationship between AQP4 and cytotoxic and/or vasogenic edema following primary injury over time. AQP4 appears to facilitate cytotoxic edema while aiding the clearance of vasogenic edema at later stages.

There is a need for clarification between the differences in the role of AQP4 in different types/stages of edema formation and resolution in different models of SCI and over the extended time-course after injury (Figure 3). Such information would also require an understanding about the impact of the severity of the injury on the role of AQP4. Furthermore, clarification is required with regard to the cellular mechanisms of regulation for AQP4 activity in the context of neurotrauma both at an expression and protein level. It is reasonable to suggest that due to the conflicting evidence in AQP4 protein/expression levels and the functional impact of such, the effect of AQP4 is mediated by its subcellular localization within the cells. 

### 3.3. AQP9 in SCI

Currently, there is only one study in rats that shows upregulation of AQP9 after SCI with myelotomy reducing SCI-induced AQP9 and correlating with reduced edema and enhanced functional recovery [130]. However, the study is confounded by the same changes in AQP4, expressed at greater levels after SCI and, hence, it is difficult to tease out the actual contribution of AQP9 in edema formation after SCI. Nonetheless, the study does show regulation of AQP9 after SCI and myelotomy and, therefore, requires further study with specific AQP9 inhibitors to dissect out the contribution of AQP9 to spinal cord edema after injury.

### 3.4. AQPs as a Clinical Target to Reduce Neurotraumatic Edema: Past, Present, and Future

The approach to treating SCI is complex one due to the highly variable nature of the condition. Despite our increasing pathophysiological knowledge of SCI including the primary, secondary, and tertiary damage mechanisms and their consequences, current therapeutic approaches remain largely palliative. This includes pain relief, rehabilitating symptomatic consequences such as muscle spasticity or bowel problems, and/or implementing methods of patient self-care for symptoms [131,132]. While these are essential elements for therapy, a large niche remains in that currently, very little can be done in the way of acute treatment to prevent or minimize the impact of secondary injuries such as edema and encouraging CNS regeneration of damaged nerves. Such therapeutics could minimize the need for existing palliative care, decrease the costs required to treat SCI patients, and could provide a higher quality of life for patients going forward.

The realization that edema can be a secondary damage-causing consequence of the initial injury is a relatively new one and, therefore, therapeutic interventions are few [115]. Currently, there is no specific therapeutic agent able to alter the movement of water through AQPs except mercury, which inhibits passage of water by disorganizing the structure of a number of oxygen-containing motifs and causes the collapse of the arginine region and physically restricts water flow [133]. The AQP water channel pores are very small and allow them to specifically permit the passage of water while restricting passage of larger molecules. As such, designing non-mercurial clinical agents able to specifically block or alter the biochemistry of these pores is a great challenge. Furthermore, the high structural conservation between members of the AQP family means that targeting specific isoforms is difficult [134]. 

Many attempts have been made at altering the genetic expression of AQP proteins to observe changes in structure and function of the CNS both physiologically and pathologically [119,135]. For example, altering protein expression is often relatively long-lasting in effect and, as discussed, it appears as though the role of AQP4 in SCI pathogenesis is biphasic in its contribution to formation and resolution of edema. Hence, knocking out AQP4 may be beneficial for preventing formation of edema in the acute stages of SCI, but the AQP4 protein must then later continue be expressed to ensure its physiological functions are maintained [136]. However, one recent study demonstrated in a mouse model of juvenile traumatic brain injury (jTBI) that siRNA treatment transiently knocks down AQP4, decreases brain edema, and improves cognitive outcomes for as long as two months post-injury [137]. However, the timescale over which the siRNA operates until it becomes degraded has not been reported. While the use of siRNA as therapeutic interventions is extremely promising on a molecular level, it remains limited in its clinical practicality due to a number of neurobiological hurdles in therapeutic administration. These include the restrictions imposed by blood-CNS barriers, the stability of the siRNA as biological molecules, and the variation in the genetics of individuals [138]. Given the apparently biphasic nature and lack of current clarification over thresholds of timepoints by which AQP4 plays its various role, perhaps shorter-term interventions are the key for future interventions.

Currently, the only drug used clinically in treatment following SCI is methylprednisolone (MD). When it was first investigated, it was considered to be an inhibitor of lipid peroxidation and was, therefore, considered to be beneficial following SCI by limiting secondary damage through this mechanism [139]. However, it has since demonstrated very little therapeutic effect throughout clinical trials with many arguing that any benefits of the drug do not outweigh the adverse effects reported such as pneumonia and respiratory failure [140,141]. Furthermore, administration of MD following contusion SCI appeared to increase the spinal cord water content alongside reducing the expression of AQP4 [142]. This suggests that, despite the potential therapeutic benefits offered to SCI patients by way of reducing lipid peroxidation, it may undo its own beneficial effect by enhancing the levels of edema. However, the mechanism by which MD affects AQP4 is not known and, therefore, the results observed may simply be just the correlation.

Another drug currently under investigation for the treatment of SCI edema is melatonin. Two earlier experimental studies suggested that melatonin was beneficial for treating SCI due to its apparent ability to reduce free radical damage and neutropil toxicity [143] and reduce lipid peroxidation [144]. Later studies focusing on SCI edema showed that, by contrast to the use of MD, melatonin treatment immediately following a clip compression SCI appears to significantly reduce both spinal cord water content and AQP4 expression after 24, 48, and 72 h following the injury [145]. Furthermore, similar compression models treated with melatonin demonstrated significantly improved scores on a number of behavioral tests including rearing open-plane field tests, Basso, Beattie and Bresnahan (BBB) locomotor tests, and the inclined plane test [146,147,148]. However, the mechanisms by which melatonin has this effect on edema and whether it is the effect of reducing edema that improves behavioral outcomes are not yet known. 

Some of the clues for pharmacologically treating cytotoxic edema acutely may still reside in biochemical studies of AQP4 and its regulation. In the CNS, the activity of astrocytic AQP4 can be regulated by both short-term and long-term mechanisms. Such short-term mechanisms include the “gating” of activity, which is controlled by phosphorylation of serine residues. PKC-mediated phosphorylation of Ser^180^ has been reported numerously to result in the inhibition of the AQP4 channel [44,149]. However, no structural changes to the AQP4 or the orthogonal array organization was observed following Ser^180^ phosphorylation [150]. Furthermore, studies have also demonstrated that phosphorylation at Ser^111^ via CaMKII and PKG results in the increased activation of AQP4 in cultured astrocytes [151]. However, the precise role and consequence of phosphorylation in AQP4 regulation remains unclear. Some evidence suggests that AQP4-transfected and V1_a_-transfected oocytes have reduced water permeability in response to Vasopressin [152] while previous evidence suggested that Vasopressin results in increased CaMKII activation in astrocytes [153], which should lead to increased AQP4 activity. As such, the role of phosphorylation as a regulatory mechanism for AQP4 activity in the CNS remains to be clarified as well as the conformational changes that occur to result in ‘gating’. 

Another short-term regulatory mechanism of interest in CNS astrocytes is that of AQP4 ‘trafficking’, which is similar in principle to the trafficking of kidney AQP2 in response to Vasopressin. Earlier studies in gastric cells demonstrated that AQP4 could be shown to be internalized and subsequently recycled to the surface membrane in response to Histamine [154]. More recently, it has been described in primary cortical astrocytes that tonicity changes in the local environment, which is likely reflective of an injured state following CNS injury, results in reversible translocation of AQP4 to and from the cell membrane. This trafficking was demonstrated to be the result of a Ca^2+^-Calmodulin (CaM)-PKA signaling cascade leading to Ser^276^ phosphorylation of AQP4, which triggers vesicular translocation between intracellular stores and the cell surface membrane [155]. Therefore, efforts to transiently inhibit the translocation of AQP4 acutely following a primary injury may offer a resolution for the prevention of cytotoxic edema while not affecting the protein levels required for clearance of vasogenic edema at later stages. Avoiding the use of genetic modification, temporary pharmacological inhibitors of these mechanisms would provide the ability to treat patients acutely and, for a period of time, required to delay the onset of cytotoxic edema until the local injury environment resolves and no longer promotes it.

## 4. Conclusions

This review highlights some of the current findings about AQPs and their regulation after SCI. It is evident that AQP1, 4 and 9 play a major role in water regulation in the spinal cord but differential expression of AQPs in different cell types suggests multiple functions that require greater understanding prior to the development of therapies aimed at reducing SCI-induced edema. For example, it is evident that AQP4 is probably the most important molecule in regulating water in the spinal cord. However, knockout mouse studies have suggested that complete deletion of AQP4 while reducing acute edema and improving functional outcomes may be detrimental in the chronic stages where AQP4 is required for the clearance of water. Hypoxia and hypertonicity clearly regulate different AQPs and, therefore, may be important mediators of AQP function. Hence, greater understanding of the biology of AQPs after SCI will lead to more effective treatments for SCI-induced edema and relief from the secondary detrimental effect. 

## Figures and Tables

**Table 1:** Comparison of expression and known or suggested functions of AQPS 1, 4 and 9 in the spinal cord and brain. DRG = dorsal root ganglia, DH = dorsal horn, CSF = cerebrospinal fluid, CPE = choroid plexus epithelia, (*) = not fully investigated.

	Spinal Cord		Brain		References
	Location	Function	Location	Function	
AQP1	Unmyelinated sensory fibers in DRG, DH, and grey matter	Pain processing	Ependymal cells in the CPE	CSF production	[31,50,52,53,54,55,56,57,58,59,60,61,62,63]
	Endothelial cells within glia limitans	Unknown	Perivascular astrocytes in white matter, and glial limitans	Cell migration, water homeostasis	
	Astrocytes within glia limitans, dorsal horn, central canal and white matter	Cell migration, water homeostasis	Neurons surrounding pial blood vessels	Axonal elongation	
	Ependymal cells within glia limitans and central canal	CSF production (*)			
AQP4	Astrocyte end-foot processes encircling capillaries in grey and white matter	Water homeostasis, ionic homeostasis	Perivascular end-foot processes in white matter	Water and waste clearance Neuronal excitability	[50,61,62,64,65,66,67,68,69,70,71,72,73,74,75,76,77,78,79,80,81]
	Astrocyte end-foot processes enveloping myelinated axons and axonal synapses	Regulation of perisynaptic volume			
	Astrocyte processes facing glia limitans and surrounding central canal	Water homeostasis (*)	Perisynaptic astrocyte end-foot processes	Synaptic function Perisynaptic volume Synapse plasticity K^+^ homeostasis	
	Fibrous astrocytes	Unknown			
	Ependymal cells within glia limitans	CSF production (*), water homeostasis (*)	Subpial and subependymal astrocyte processes	Water flow	
			Muller cells	K^+^ clearance	
AQP9	Astrocyte end-foot processes in white matter and glia limitans	Water flow (*)	Catacholinergic neurons	Energy metabolism (*)	[50,62,82,83]
			Astrocytes in glia limitans	Water flow (*)	
